# Biomimetic Sensors for the Senses: Towards Better Understanding of Taste and Odor Sensation

**DOI:** 10.3390/s17122881

**Published:** 2017-12-11

**Authors:** Chunsheng Wu, Ya-Wen Du, Liquan Huang, Yaron Ben-Shoshan Galeczki, Ayana Dagan-Wiener, Michael Naim, Masha Y. Niv, Ping Wang

**Affiliations:** 1 Institute of Medical Engineering, School of Basic Medical Science, Health Science Center, Xi’an Jiaotong University, Xi’an 710061, China; wuchunsheng@xjtu.edu.cn; 2 College of Life Sciences, Zhejiang University, Hangzhou 310013, China; ZQsBao@163.com (Y.-W.D.); huangliquan@zju.edu.cn (L.H.); 3 Monell Chemical Senses Center, 3500 Market Street, Philadelphia, PA 19104, USA; 4 The Institute of Biochemistry, Food Science and Nutrition, The Robert H. Smith Faculty of Agriculture, Food and Environment, The Hebrew University of Jerusalem, Rehovot 76100, Israel; yaronbsg@gmail.com (Y.B.-S.G.); Ayana.Wiener@mail.huji.ac.il (A.D.-W.); michael.naim@mail.huji.ac.il (M.N.); 5 Biosensor National Special Laboratory, Key Laboratory for Biomedical Engineering of Ministry of Education, Department of Biomedical Engineering, Zhejiang University, Hangzhou 310027, China

**Keywords:** biomimetic sensors for senses, taste sensation, odor sensation, olfaction, biosensors, chemical sensing, signal transduction

## Abstract

Taste and smell are very important chemical senses that provide indispensable information on food quality, potential mates and potential danger. In recent decades, much progress has been achieved regarding the underlying molecular and cellular mechanisms of taste and odor senses. Recently, biosensors have been developed for detecting odorants and tastants as well as for studying ligand-receptor interactions. This review summarizes the currently available biosensing approaches, which can be classified into two main categories: in vitro and in vivo approaches. The former is based on utilizing biological components such as taste and olfactory tissues, cells and receptors, as sensitive elements. The latter is dependent on signals recorded from animals’ signaling pathways using implanted microelectrodes into living animals. Advantages and disadvantages of these two approaches, as well as differences in terms of sensing principles and applications are highlighted. The main current challenges, future trends and prospects of research in biomimetic taste and odor sensors are discussed.

## 1. Introduction

Taste and odor sensations play crucial roles in most creatures by enabling recognition of chemical signals that provide indispensable information for evaluating food quality, searching potential mates and detecting danger [1,2]. The biological taste and olfactory systems are able to sense chemical signals such as tastants and odorants in complex environments with extremely high performances, unmatched by any current artificial devices. In recent decades, fast advances in elucidations of biological mechanisms of olfactory and taste sensation have been achieved. Functional components responsible for chemical sensing have been discovered, including olfactory and taste receptors, cells and tissues, which are able to detect and transduce chemical signals into biological signals such as generating neuronal action potentials and releasing neurotransmitters [2,3]. For instance, the gene family encoding vertebrate olfactory receptors (ORs) has been discovered, which has greatly promoted the basic research on molecular mechanisms of odor sensation [2]. Likewise, the receptors for sweet [4] and bitter [5] taste were identified. However, the underlying mechanisms of taste and odor sensation have not been completely understood. The chemical space of the ligands remains largely unexplored and the chemical features of ligands able to act on multiple receptors need further characterization. This is challenging, due to the large number of receptors and a complex matrix of interactions between tastants and odorants with their respective receptors. 

Biosensors as an emerging and promising technology provide new approaches for the research of taste and odor sensation. These can be classified into two main categories: in vivo and in vitro approaches and are the focus of the current review. Firstly, biological mechanisms of taste and odor sensation are introduced briefly. Secondly, the biosensors based on in vitro and in vivo approaches are discussed in detail. In vitro biosensing approaches are outlined based on the biological functional components utilized as sensitive elements including taste and olfactory tissues, cells and receptors. In vivo biosensing approaches are introduced focusing on the extracellular recordings by implantable microelectrodes towards taste and olfactory signaling pathways into living animals. These biosensing approaches are compared based on their sensing principles and applications. Advantages and disadvantages of these biosensing approaches are examined and the current main challenges, future trends and prospects of research in taste and odor sensation are discussed. 

## 2. Biological Mechanisms of Taste and Olfaction Sensation

**Figure 1:**
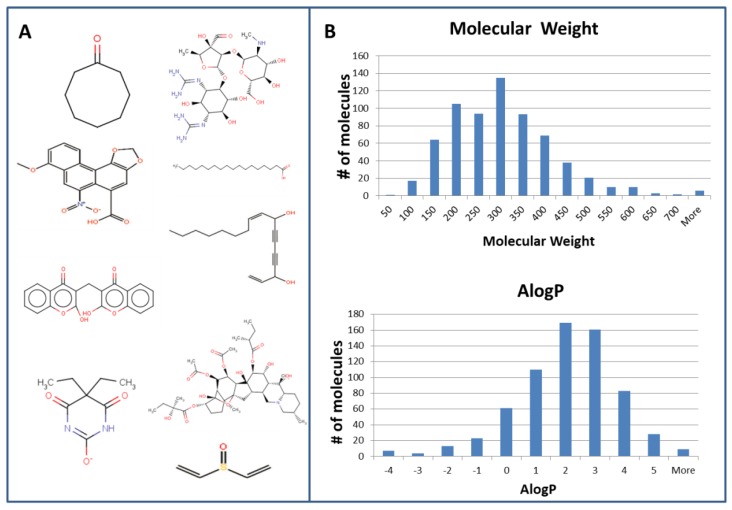
Bitter molecules are very diverse in their chemical structure and physicochemical properties. (**A**) Examples of molecules from variety of chemical families which were reported to elicit bitter taste; (**B**) Distribution of MW and AlogP values calculated for BitterDB molecules.

Gustation or taste is an indispensable physiological sensation, which is essential for taste evaluation, as well as food choice and consumption [2]. It is now becoming clear that taste receptors play also extra-oral roles [6] and are considered as novel therapeutic targets [7]. Thus, identifying new taste compounds, as well as detecting presence of taste compounds in food, drug candidates and drug formulations is of foremost importance for quality and palatability monitoring of food, as well as for discovery and formulation of new drugs. There are five basic taste qualities (sour, sweet, bitter, salty and umami) [8]. Bitter is perhaps the most complex one, due to multiple bitter tastants that activate multiple bitter taste (T2R) receptor types [9]. This situation somewhat resembles the enormous complexity of smell perception, with hundreds of olfactory receptor types [10], although a much smaller number of receptor types are involved in bitter taste recognition. Currently about 700 compounds are known to be bitter to humans, as summarized in the BitterDB database [9]. Analysis of bitter compounds and bitter receptors has suggested that bitter ligands that are recognized by multiple bitter receptors are typically small globular molecules from natural origin, while those that are recognized by few ones are large and flat molecules, many of which are synthetic [11]. Currently available data suggests that promiscuity of ligands towards bitter taste receptors is similar across different species but different from general promiscuity towards protein targets. Recent analysis also shows that T2Rs have exceptionally high agonist to antagonist ratio (i.e., easier to be activated than inhibited) and typically have relatively lower affinity towards their ligands compared to other members of the GPCRs superfamily [12]. Although bitter compounds are traditionally considered hydrophobic, an inspection of BitterDB shows that hydrophobicity and other physicochemical properties of bitter compounds are rather variable, as can be seen in Figure 1, which was prepared based on [9].

Taste plays a crucial role in ingestion, by providing crucial information about nutritive (carbohydrate or protein rich) or toxic nature of potential food. Bitter taste is typically considered to be a marker of toxicity. However, the relation between bitterness and toxicity is not a simple one, as recently shown by Nissim et al. [13]. Furthermore, Niv and coworkers have shown that while many bitter compounds that activate T2R14 are also human Ether-a-go-go Related Gene (hERG) inhibitors, certain Traditional Chinese Medicine (TCM) compounds [14] that are predicted to activate the same bitter taste receptor, are less frequently hERG inhibitors [15]. Since hERG, a potassium channel, has a critical role in cardiac action potential repolarization, compounds inhibiting its function can cause lethal arrhythmias and are thus deemed toxic. Identification and characterization of active ingredients from TCM can shed light on the mechanisms of TCM medicinal functions. Many compounds that have been isolated from the Chinese *materia medica* exhibit pharmacological activities. The most striking example is artemisinin, as celebrated by the 2015 Nobel Prize for Youyou Tu for her discovery of its role in treating malaria. Notably, many pharmaceuticals taste bitter [15,16]. Bitter-tasting compounds may have specific physiological effects in T2R-expressing cells. Traditional Chinese drugs are classified according to “four natures” (si qi; hot, warm, cool, cold or neutral) and “five flavors” or “five tastes” (wu wei; acrid/pungent, sweet, bitter, sour and salty). Thus, mining and exploring the TCM compounds (compiled in the TCM database [14] and available via the ZINC database [17]) for bitterness computational tools such as BitterPredict [18,19] can help understand the actions of diverse TCM compounds and could reveal potential therapeutic values. 

**Figure 2:**
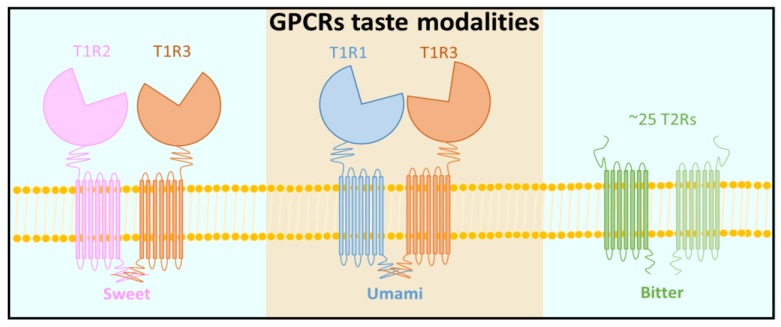
Receptor components for sweet, umami and bitter tastes.

In addition to bitter taste quality, there are other four basic taste modalities including sour, sweet, salty and umami. Similar to bitter sensation, sweet and umami tastants are recognized by specific G-protein coupled receptors (GPCRs) located on the taste cell membrane. The receptors involved in mammalian sweet, umami and bitter taste sensation are summarized in Figure 2. These signals are subsequently mediated by second messengers, such as cAMP, IP_3_ and calcium [20]. T1R1, T1R2 and T1R3 are responsible for the detection of sweet and umami and belong to the class C GPCRs, which have a large extracellular domain (ECD) containing a Venus flytrap (VFT) domain that serves as the orthosteric binding site for typical ligands [21]. The T1R2 and T1R3 form a heterodimer (T1R2+3) that functions as sweet taste receptor for the detection of sweet tastants such as sugars, synthetic sweeteners and sweet tasting proteins, while T1R1 and T1R3 form a heterodimer (T1R1+3) that functions as umami taste receptor for the detection of umami stimuli [4,22,23,24]. Sour and salt taste signals are detected by ion channel receptors. In recent decades, several ion channels have been reported to serve as molecular sensors for sour signal detection, which include acid-sensing ion channel 2 (ASIC2), hyperpolarization-activated cyclic nucleotide-gated channels (HCNs), two-pore domain K^+^ channels and polycystic kidney disease-like (PKD) channels [25,26,27,28,29] but no consensus about the main sourness sensor has been reached so far [2]. The amiloride­sensitive epithelial Na channel (ENaC) is a heterotrimeric protein that consists of three subunits: α, β and γ in rat and mouse in which it is reported to sense salt [30]. 

Smell is another key chemical sense, which enables recognition and discrimination of a large number of distinct odors and plays an important role in most animals’ life qualities. In most cases, the sense of smell monitors food and environmental odors by employing the main olfactory epithelium (MOE), which provides important information about food and potential dangers (e.g., smoke) [31]. On the other hand, in many animals, vomeronasal organ (VNO) is sensitive to potential interpersonal chemosignaling (e.g., sexual selection), which is very important for mate-searching [32]. Since the discovery of the gene superfamily encoding olfactory receptors, research on mechanisms of odor sensation has achieved significant progress in the field [1,33]. It was demonstrated that olfactory receptors, which belong to members of the GPCR superfamily, are located in the cilia of olfactory receptor neurons (ORNs) and able to bind specific odorant molecules and initiate a cascade of intracellular enzymatic reactions [34], via second messengers [33]. These intracellular reactions result in the generation of action potentials, which are transmitted to the olfactory bulb for further processing and finally arrive at the olfactory cortex for deciphering olfactory signals, leading to the recognition and discrimination of distinct odors [35]. This process realizes the conversion of chemical signals of odorants into electrophysiological signals of neuronal systems, which finally allows for the perception of odorants. It is worth to note that the threshold for taste is typically much higher than for olfaction. For example, beta-damascenone, an odorant that appears in various fruits and flowers, has odor detection threshold of around 10^−13^ M [36]; 2-methyl-3-furanthiol (MFT), a sulfur odorant that in most cases is aversive to humans, has odor detection threshold of around 10^−14^ M [37]. On the other hand, thresholds for bitter tastants are several folds higher, i.e., for caffeine around 10^−6^ M and for propylthiouracil around 10^−7^ M [38].

To facilitate research on taste and smell, databases of known and predicted ligands have been established. Specifically, the aforementioned BitterDB gathers bitter ligands [9]. SuperSweet gathers known and predicted sweet compounds [39]. Olfactory receptor databases were established SenseLab (http://senselab.med.yale.edu/ordb) and HORDE (The Human Olfactory Receptor Data Explorer, http://genome.weizmann.ac.il/horde/). SuperScent is a database of flavors and scents [40].

Sensors refer to devices that are able to transform non-electrical responsive signals into electrical signals that are the most convenient for processing, transportation, display and recording. Sensors usually provide information about the physical, chemical or biological state of a system. Sensors are often composed of sensitive elements, conversion components and related electronic circuits. Fast advances in microfabrication process and sensing technologies promote the research of artificial sensors for taste and smell that are able to mimic mechanisms of taste and smell, which will be briefly introduced in Section 3. Biomimetic sensors for taste and odor sensation have achieved fast progress, in which biological functional components such as tissues, cells and receptors, are employed as sensitive elements for chemical sensing. This will be briefly introduced in Section 4. Section 5 and Section 6 highlight recent work with in vitro and in vivo biosensing approaches for the chemicals senses, respectively. Section 7 summarizes possible ways for further improvement.

## 3. Artificial Sensors for Taste and Smell

In artificial biosensors, these sensitive elements are usually coupled with various transducers that are able to transduce the sensing signals of sensitive elements into electrical signals. Many kinds of transducers have been utilized for the development of olfactory-based biosensors such as quartz crystal microbalance (QCM), field-effect transistor (FET), microelectrode, surface plasmon resonance (SPR) and light-addressable potentiometric sensor (LAPS) [41,42,43,44,45,46]. In recent decades, various approaches have been applied in the research of odor sensation mechanisms, which could be utilized to investigate olfactory transduction mechanisms in comprehensive aspects and provide deep insight into the underlying mechanisms of odor sensation. Human olfactory receptor responses to odorants were recently studied [47] and multiple odorant-receptor pairs are currently known. While much effort is still required to find ligands of the multiple remaining orphan (i.e., having no known ligand) ORs, the chemical space of the deorphanized ORs can be readily explored by combined computational and in vitro approaches, as described in [48]. The combinations of olfactory cells or receptors with micro/nano sensors or devices allowed the development of bioelectronic noses [49,50]. Screening with biomimetic sensors: e-noses and e-tongues based on two main sensors, potentiometric or voltammetric sensors arrays for multicomponent analysis [51]. E-tongues have been applied in a large number of fields including food quality monitoring [52] and environmental protection [53]. However, the sensitivity, specificity and response speed of current e-noses and e-tongues are still inferior to those of biological olfaction and taste systems. Today, due to advances in understanding of biological mechanisms of chemosensation, various sensors are being developed by bio-mimicking taste and smell signal transduction pathways for applications in food safety, biomedicine, environmental protection and terrorism prevention [54]. Odor visualization [55] and standardization [56] are expected to be of practical importance for general vapor dosimeters and analyte-specific detectors. Although much progress has been obtained, further work, based on the combination of multiple disciplines such as biology, electronics and information technology is necessary. Current difficulties include the identification of specific olfactory receptor-ligand pairs, the characterization of various olfactory receptors, the mechanisms of encoding and decoding of olfactory signals. Meanwhile, the development of olfactory-based biosensors also faces some critical challenges such as the preparation of functional biological components, enhancement in the sensitivity, reproducibility and realization of high-throughput and multivariate analysis. In addition, the stability and repeatability of the olfactory-based biosensors constructed based on olfaction-inspired materials are still limited. To address these issues, olfactory-based biosensors characterized with micro/nano sensor arrays, multiple responsive capabilities and user-friendly interfaces will be the main trends in the near future. 

## 4. Biomimetic Sensors for Taste and Odor Sensation Mechanisms

The possible ways of identifying (or confirming computationally predicted) agonists of chemosensory receptors include: (i) screening of cells that heterologously express a single taste receptor [14,18,57]; (ii) quantifying licking or consumption animal tests [58,59]. This latter behavioral testing method relies on the similarity of the taste systems in different organisms. Yet, such similarity is only partial because of different number of T2Rs and partial overlap in ligand recognition. For sweet taste, for which both mice and humans have only one sweet taste receptor, the sensitivity to sweet compounds varies dramatically [60,61], while chicken lack T1R2 monomer altogether [62]. Awareness and understanding of the species-dependent differences in preference testing for novel tastants are critical for the methods using animal models of taste and nutrition.

Biosensors, as an emerging approach, are increasingly applied in the research of taste and smell sensing mechanisms and studies of ligands and modulators, which are commonly developed by the combination of biological functional components originated from biological taste and olfactory systems with various transducers [63,64]. These biosensors can be mainly classified into two categories, which are in vitro biosensing approaches and in vivo biosensing approaches. The biosensors usually show high performances for chemical sensing and biochemical analysis. For example, high sensitivity and rapid response can be achieved in biosensors due to the utilization of biological functional components such as taste and olfactory receptors and cells. Moreover, computational neural network models have also been applied together with biosensing approaches for improving the performances of these biosensors. For instance, K serial models, a non-linear neuronal network model based on the theory of nerve cell groups, have been utilized as building blocks of the neuronal networks at different anatomical levels of olfactory systems [65]. The advances in the research of taste and smell sensing mechanisms can drive the development of taste- and smell-based biosensors. For example, the lipid-polymeric membrane-based biosensors for taste sensing are developed based on the discovery of mechanisms of taste cell membrane in taste sensing [66]. These biosensors provide powerful and accurate approach for the research of taste and odor sensation mechanisms. However, the sensitivity and reproducibility of current biosensors are still limited due to the special properties of sensitive elements originated from biological taste and smell systems as well as the complexity of the underlying mechanisms. 

## 5. In Vitro Biosensing Approaches

The basic idea of in vitro biosensing approaches is using biological functional components isolated from biological taste and smell systems as sensitive elements to couple with various transducers for converting responsive signals into optical/electrical outputs. The biological functional components could be tissues (e.g., taste buds, olfactory epithelium), cells (e.g., taste receptor cells, olfactory sensory neurons) and receptors (taste receptors, olfactory receptors), which usually maintain the central mechanisms and characters of natural chemical sensing capabilities. 

### 5.1. Tissue-Based In Vitro Biosensing Approaches

**Figure 3:**
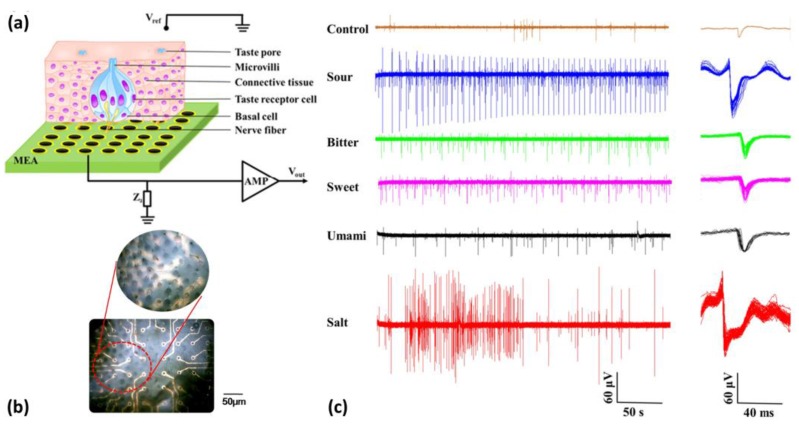
(**a**) Schematic diagram of in vitro biosensing approach using taste epithelium coupled with MEA chip; (**b**) Image of taste epithelium coupled with MEA chip; (**c**) Recorded typical responsive electrophysiological signals upon the stimulations of five basic taste qualities [67,68].

Taste buds are special taste sensation organs located in taste epithelium and are composed of different types of taste cells responsible for the detection of taste signals presented by various tastants. Taste epithelium isolated from rats has been utilized as sensitive elements for taste signal transduction, when coupled with a microelectrode array (MEA) that is able to record responsive taste electrophysiological signals upon taste stimulations [67,68,69]. Figure 3a illustrate taste tissue-based in vitro biosensing approach using MEA chip for extracellular recording. Figure 3b is image of taste epithelium coupled with MEA chip. Figure 3c shows the typical responsive electrophysiological signals from taste epithelium isolated from rat under stimulations of five basic taste qualities, from which distinct spatiotemporal patterns could be observed. In addition, dose-dependent electrophysiological signals could be obtained under the stimulation of bitter substances at different concentrations. 

Similarly, olfactory epithelium is the special organ for odorant detection, which contains various types of olfactory receptor neurons that are able to transduce the chemical signals to cellular responses including action potential changes. Olfactory epithelium isolated from rats has been coupled with MEA for recording responsive olfactory signals under odorant stimulations [70,71]. MEA was able to perform parallel multi-site extracellular recordings, which could be used for spatio-temporal analysis. Different odorant stimuli elicited different firing modes as indicated by the analysis on the electrophysiological recordings under stimulations of ethyl ether, acetic acid, butanedione and acetone. In the case of isoamyl acetate or l-carvone stimulation, the frequency of spiking activity responded in a concentration-dependent manner [71]. LAPS has also been employed to record the electrophysiological signals from olfactory epithelium in response to acetic acid and butanedione, from which the different frequencies and firing modes as well as the characteristic frequency peaks corresponding to these two odorants were recorded [72]. Antennal olfactory sensilla that are specialized organs found in many insects for sensing environmental chemical compounds were also utilized as sensitive elements for the detection of specific odorants [41,73]. Isolated antennae were coupled with FET via an electrolyte solution, which is named isolated antennae method. The reference electrode is directly connected with the insect antennae. Field-effect devices (FEDs) were used as transducers to record the depolarization responses evoked by specific odorant stimulation from antennal olfactory sensilla. The measurement results show that (Z)-3-hexen-1-ol, a volatile biomarker for plant damage, could be detected ranging from 0.1 parts per million (ppm) to 100 ppm, which have great potential to be applied in the monitoring plant damage in a greenhouse. Another approach is the whole insect method in which insect antennae are still kept in the body of insect and fixed on the surface of transducers. For example, an intact pyrophilic beetle was coupled with FEDs to serve as sensitive elements for the detection of specific chemical volatile compounds [74].

### 5.2. Cell-Based In Vitro Biosensing Approaches

Functional taste and olfactory cells that suitable to be used as sensitive elements for chemical sensing can be obtained from animals (i.e., primary cells) or bioengineered approaches. Primary cells are usually directly isolated from animals, while bioengineered cells can be obtained by the expression of specific taste or olfactory receptors in a heterologous cell system. In case of the OR preparation, the most frequently used expression systems include human embryonic kidney (HEK-293) cells and Michigan Cancer Foundation (MCF)-7 cells. The preparation of primary cells is relatively convenient but limited in applications due to the difficulties in identification and characterization of different types of receptors in the desired cells. In contrast, bioengineered cells offer well-defined types of receptors and sensing capabilities. Functional cells obtained from both approaches have been employed in the research of taste and smell modulators. 

**Figure 4:**
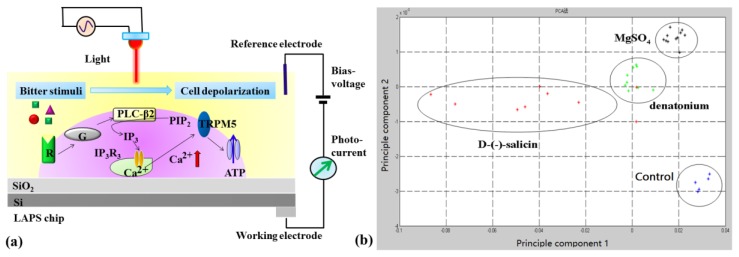
(**a**) Schematics of LAPS cultured with taste receptor cells for the detection of specific bitter substances; (**b**) Principal component analysis (PCA) results indicate that different bitter substances could be discriminated by these taste receptor cell-based biosensors, reproduced from [78].

Taste receptor cells have been coupled with various transducers and served as sensitive elements for the detection of different taste signals. For example, primary taste receptor cells isolated from rat have been cultured on the surface of LAPS, which is able to record cellular responses from single taste receptor cells under various tastant stimulations [75,76,77,78]. Multiple taste compounds including NaCl, HCl, MgSO_4_, sucrose and glutamate, could be distinguished by monitoring the extracellular potential changes of the taste receptor cells [75]. The temporal firing responses recorded from different types of taste receptor cells were different and the firing rate was dependent on the concentrations of tastants [76]. It is interesting that both enhancive and inhibitory effects of exogenous adenosine triphosphate (ATP) on the spontaneous firing of taste receptor cells could be observed using this in vitro biosensing setup. Specifically, this kind of taste receptor cell and LAPS hybrid biosensors have been applied for acidic sensation and the detection of bitter substances [77,78]. Figure 4a shows the basic principle of LAPS cultured with taste receptor cells for the detection of specific bitter substances. As shown in Figure 4b, the measurement results indicate that this taste cell-based biosensor was able to discriminate different bitter substances based on the processing of LAPS extracellular recording signals using principal component analysis (PCA). Cells expressed with PKD channels that are members of transient receptor potential ion channels were coupled with MEA and used for acid sensation, which was able to record the special off-responses of PKD channels under acid stimulations [79]. Similarly, cells expressing the sweet taste receptor T1R1/T1R3 were coupled with electrochemical sensors for the detection of different concentrations of sucrose [80]. Furthermore, cells expressed type 2 member receptors (T2Rs) were used as recognition elements and coupled with electrochemical sensors for the detection of different concentrations of bitter substances including quinine, *N*-Phenylthiourea and 6-propyl-2-thiouracil [81,82]. An additional novel sensor was constructed based on murine sperm cells by the Wang group and a range of bitter compounds were tested [83,84]. It became clear that enhancement of sensitivity of biomimetic sensors is essential.

**Figure 5:**
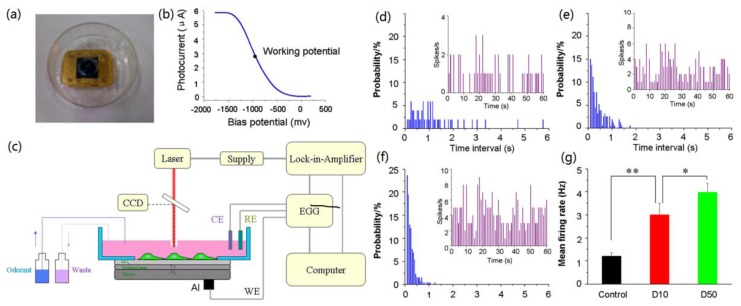
(**a**) LAPS chip with a detection chamber; (**b**) The typical LAPS *I-V* curve (photocurrent vs. bias potential); (**c**) A schematic diagram of LAPS measurement setup; The temporal response mode recorded by LAPS from bioengineered olfactory receptor neurons under conditions of (**d**) spontaneous firing, i.e., control; (**e**) 10 mM diacetyl stimulation; and (**f**) 50 mM diacetyl stimulation; (**g**) Mean firing rate statistics of control, 10 mM diacetyl (D10) stimulation and 50 mM diacetyl (D50) stimulation [87].

Similar to taste cells, primary olfactory cells isolated from rats have been coupled with LAPS for the detection of odorants based on the special capability of LAPS on extracellular recording from single cells [85,86]. The measurement results indicated that different odorants elicit different firing spikes and the enhancive and inhibitory effects on olfactory signals could be observed, which demonstrate the origination of LAPS recording are from olfactory signal transduction. Bioengineering primary olfactory cells expressed with ODR-10, which is an olfactory receptor of *C. elegans* specific to diacetyl, were coupled with LAPS for the detection of diacetyl [87]. Figure 5a–c shows the mechanism and setup for in vitro biosensing approach using LAPS chip coupled with bioengineering primary olfactory cells. LAPS recording results show that different firing patterns were elicited using low/high concentrations of diacetyl (Figure 5d–g). In addition, dose-dependent responsive signals were obtained in the amplitude patterns of the temporal firing spikes within the concentration of diacetyl ranging from 0.1 μM to 100 μM. In addition to LAPS, MEA has also been utilized to record the cellular responses from primary olfactory receptor neurons under the stimulation of odorants [88]. Similarly, a microfabricated planar electrode was applied to monitor the membrane potential changes of the cells expressing olfactory receptor I7 and gustatory cyclic nucleotide gated (CNG) channel, which were used to detect the specific odorant, octanol and to amplify the membrane potential, respectively [89]. Furthermore, glass capillary Ag/AgCl electrodes were couple with microfluidic chip to monitor the responses of cells expressing insect olfactory receptors under the stimulations of different odorants [90]. SPR technique has also been applied in the detection of responsive signals from bioengineered cells expressing the specific olfactory receptor, ODR-10, under the stimulation of diacetyl that is a natural ligand of ODR-10 [91].

### 5.3. Receptor-Based In Vitro Biosensing Approaches

**Figure 6:**
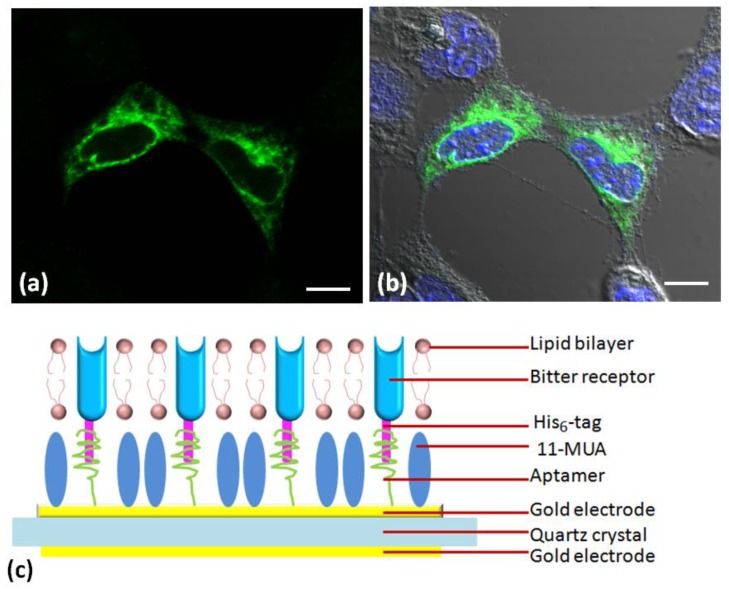
Expression of taste receptors in HEK-293 cells indicated by (**a**) fluorescent image and (**b**) combination of fluorescent and bright-field images; (**c**) Schematic diagram of taste receptors coupled with the gold electrode surface of QCM devices via self-assembled aptamers [94].

Taste and olfactory receptors are molecular detectors for chemical signals, which are able to directly interact with target ligands. These receptors have been coupled with different transducers for the development of receptor-based biosensors for the research of taste and odor sensation mechanisms. The receptors used as sensitive elements of biosensors should be produced in a cost-effect manner and maintain their natural structure and native functions in a somewhat long term. Various approaches have been reported for the preparation of receptors suitable to be utilized as sensitive elements for chemical sensing. Chemical synthetic peptides mimicking functions of ORs are also considered as biological materials for OR-based biosensors [92]. These approaches could be classified into two categories that are (1) directly extraction from living tissues/animals and (2) functional expression in a heterologous cell system, respectively. Both approaches have advantages and disadvantages. Direct extraction is convenient and cost effective but make is it difficult to identify and purify the desired types of receptors. For instance, olfactory receptor proteins isolated from living olfactory epithelium of bullfrogs have been coated onto the gold electrode surface of quartz crystal microbalance (QCM) devices to construct a QCM array for the detection of specific odorant molecules [93]. On the other hand, functional expression could overcome the shortcomings of direct extraction to some extent but still suffer from the low yield and high cost. The receptors that are expressed in a heterologous cell system are usually extracted and coupled with transducers that are able to detect the specific interactions between receptors and ligands. For example, Wang et al. have previously expressed bitter taste receptor T2R4 in HEK-293 and detected the change of the calcium concentration in these cells when stimulated by bitter compounds. The membrane fractions containing the expressed T2R4 were extracted and immobilized on the gold surface of a QCM pretreated with a monolayer of self-assembled aptamers that can specifically recognize and capture biomolecules labeled with His_6_-tags (Figure 6). The QCM device was used to monitor the responses of T2R4 to various bitter stimuli. The results indicate that this biosensor can detect denatonium with high sensitivity and specificity, which is known to be an agonist of T2R4 [94]. Similarly, olfactory receptors including the rat olfactory receptor I7 and the olfactory receptor of *C. elegans* ODR-10 have also been expressed in a heterologous cell system and extracted to couple with QCM devices for the detection of specific odorants such as octyl aldehyde and diacetyl, where the dose-dependent responses could be obtained [41,95]. 

Interestingly, a recent study has shown that amphipathic compounds can significantly amplify β2AR signaling and delay its desensitization via intracellular inhibition of G-protein-coupled receptor kinase 2 (GRK2) [96]. Furthermore, receptor activity-modifying proteins (RAMPs) provide an important example of proteins that interact with GPCRs to modify their function [97] and receptor transporting proteins (RTPs) and receptor expression enhancing proteins (REEPs) can enhance olfactory and taste receptor function [98,99]. It has been reported that co-expression of a particular G-protein subunit can significantly increase its partner’s protein level, thus markedly augmenting receptor-induced G protein-transduced signaling efficiency [100]. Such mechanisms open up new avenues for improving biosensor signaling.

**Figure 7:**
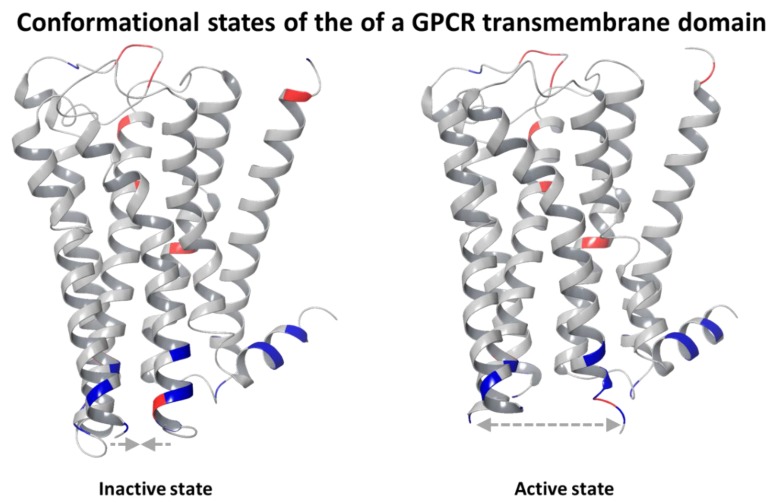
Schematic diagram illustrating the conformational change of rat OR-17 that was used to monitor ligand presented in [104].

Surface acoustic wave (SAW) devices that are mass-sensitive devices with higher sensitivity compared with QCM devices have also been applied in the development of receptor-based biosensors for the research of odor sensation. Wu et al. developed a SAW-based biosensor for highly sensitive functional assays of olfactory receptors [101]. An olfactory receptor ORR-10 was expressed in HEK-293 cells and then extracted in order to be immobilized on the sensitive area of SAW devices. The specific interactions between ODR-10 and its natural ligand, diacetyl, were monitored by recording the resonance frequency shifts of SAWs, which are proportional to the mass changes, thus reflecting the binding events of the olfactory receptor with its ligand occurred on the sensitive area of SAW devices. Furthermore, a self-assembled monolayers (SAMs)-based approach was reported in order to improve the coupling efficiency of olfactory receptors with SAW devices [102]. It is demonstrated that this approach was able to improve the coupling efficiency of olfactory receptors with SAW devices and lead to the enhancement in sensitivity by two times. In addition to mass-sensitive devices such as QCM and SAW devices, electrochemical impedance spectroscopy (EIS) has also been applied in the detection of specific interactions of olfactory receptors with odorant ligands. The rat olfactory receptor I7 (ORI7) was reported to be immobilized on a gold electrode for the quantitative odorant detection by EIS measurements that are sensitive to the conformational changes of olfactory receptors resulting from the binding effects of ligands [103]. Figure 7 is an example for an open and closed conformations of a GPCRs, which polarization resistance changes originated from the binding effects of ligands on the olfactory receptors were also able to be measured by EIS, which were indicated by the measurement on the impedance measurement on the rat receptor OR-I7 immobilized on the electrochemical sensors [104]. In addition to the measurement, the underlying mechanisms were further investigated by providing the theoretical framework that was able to predict and interpret electrical properties of a single olfactory receptor [104,105,106]. 

In summary, in vitro biosensing approaches are useful and promising for the research of taste and odor sensation mechanisms and are characterized by high sensitivity, high specificity and rapid responsiveness. However, these approaches usually suffered from the limited lifetime of sensitive elements due to the lack of efficient in vitro culture conditions to maintain the functions/activities of tissues/cells/receptors in a long term. In addition, the cell damage and death are often unavoidable during the measurement process. As a result, the working life of in vitro biosensors is usually limited to a few hours, which makes it difficult to realize long-term and repeatable measurement and therefore hamper their practical applications. 

## 6. In Vivo Biosensing Approaches

With the fast advances in the research of neural recording and decoding mechanisms, in vivo biosensing approaches attract increasing interest due to their promising potential in addressing the shortcomings and difficulties of the in vitro biosensing approaches. In addition, the rapid progress in the microfabrication process makes it possible to fabricate implantable devices that are able to significantly reduce the damage to the cells and tissues at the implanted location, which allows for the long-term and real-time in vivo measurement. The basic idea of in vivo biosensing approaches is to utilize the whole animal as a sensitive element, to record the neural activities from biological taste and smell sensing systems and to evaluate the sensing signals by neural decoding methods. At present, the most frequently used devices for recording the neural activities are implantable MEAs, which are able to be implanted into the animals following proper surgery protocols and collect the neural electrophysiological data in a long term. 

The biological taste and smell systems could be considered as a black box with tongue and nose as sensitive elements and brain regions as signal processing units. The electrical output of brain regions is recorded by implanted MEAs and used for further analysis and identification. For instance, neuronal signals from taste systems recorded by microelectrodes are usually processed by micromesh bandpass filters, which are able to split the wideband signal into high frequency part, spikes, low frequency part and local field potential [107]. Meanwhile the information of tastant delivery process can also be recorded and combined with the neuronal signals in order to adjust the physical delays between recorded signals and the time at which the tastant acted on taste organs. Multiple sensitivity of chorda tympani fibers of rats to taste stimuli have been investigated by in vivo biosensing approaches, in which impulse discharges in single chorda tympani fibers in response to four basic qualities of taste stimuli were obtained and the types of responsive units could be categorized statistically [108]. On the other hand, microelectrodes implanted into the hamster’s soft palate were applied to record the proportional responses to different taste stimulations from four different nerve cell-types in the hamster gustatory system [109]. The results indicated that the greater superior petrosal nerve, which provides most of the taste information to the brainstem about sucrose and NaCl together with CT nerve, is more responsive to sucrose. 

**Figure 8:**
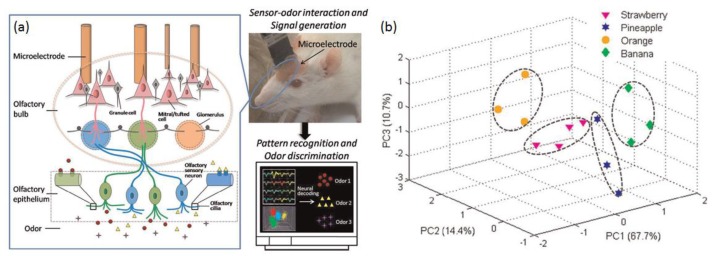
(**a**) Schematic diagram showing the configuration of electronic nose based on in vivo biosensing approaches; (**b**) The result of PCA on recorded data [122].

Some animals have very high sensitivity to odors and are able to detect thousands of odorant molecules even at trace level. For example, dogs have been trained to trace the location of dangerous and toxic odorant molecules including explosives, illicit drugs, land mines etc. [110,111]. The output of these trained dogs usually relies on the behavioral readout by a trainer and experimenter, which are usually indirect and inconvenient [112]. In vivo biosensing approaches could address this issue to some extent, by utilizing artificial devices such as MEAs to record the responsive olfactory signals and extracting from these the olfactory signals. At present, with the fast advances in the implantable MEAs, odor information can be measured through changes in the average firing rate of neurons located in olfactory bulb. In addition, in vivo biosensing approaches have been able to perform continuous extracellular recordings for up to 18 months [113,114]. Implantable MEAs composed of dozens of microelectrodes with diameters around few microns were often inserted into the tissues of biological nerve systems [115,116]. The least damage to cells and tissues at the implanted locations could be achieved by the minimally invasive implant approaches, which allows for the continuous extracellular recording of action potentials from biological olfactory systems in a long-term and repeatable manner [117,118]. The neural decoding methods could be applied to process the recorded olfactory signals in order to extract the odorant information sensed by animal olfactory systems, which allows more accurate extraction of specific patterns of olfactory neuronal activities [119]. Implantable MEAs are able to perform simultaneous multisite extracellular recordings of action potentials from the neurons located within a radius of 140 μm, containing ~1000 neurons in the rat cortex. The recorded spikes usually have amplitudes higher than 60 μV from neurons located near the microelectrode within the distance of 50 μm (~100 neurons), which makes it possible to perform clustering separation due to the identical action potentials generated by the same class of neurons [120]. Therefore, the in vivo biosensing approaches usually characterized with high sensitivity, high stability and good repeatability. For instance, an electronic nose based on in vivo biosensing approaches has been reported as shown in Figure 8, in which a 16-channel MEAs were implanted into the olfactory bulb in vivo for recording the extracellular action potentials of mitral/tufted (M/T) cells that are the output neurons of olfactory bulb [121,122]. The firing patterns as indicated by the extracellular recordings showed noticeable differences in temporal features and rate features upon the application of different odorant molecules and concentrations. 

The in vitro biosensing approaches usually benefit from the highly efficient sensor fabrication, various commercially available transducers, convenience in function modulation and the ability to provide long-term solutions. On the other hand, these approaches are costly, may encounter ethical issues (animal experiments are difficult to get approval for, or even prohibited in some areas) and face challenges in interpretation of the readouts. This field provides exciting opportunities for future developments. 

## 7. Conclusions and Prospects

Rapid advances in the biosensing technologies have promoted the research in the field of taste and odor sensation. Biosensing approaches have contributed to the exploration of taste and odor sensation, for example, the specific interactions between receptors and ligands [52,93,95], chemical sensing using olfactory/taste cells [54,63,64] and odor sensing using in vivo biological olfactory sensing systems [118,119,120,121,122]. However, these approaches still face many challenges for further applications due to their own inherent shortcomings. For instance, low response intensity caused by weak coupling between sensitive elements and transducers: sensitive elements such as cells are usually immobilized unstably in the coupling progress and thus the cellular response is lower than typical adherent cells. Chemical immobilization methods can be used to overcome this challenge by enhancing response intensity by improving coupling between cells and transducers. Other methods to increase immobilization efficiency and stability of ORs, such as physical adsorption and layer-by-layer self-assemble monolayers have also been utilized. Sensitivity and stability still need to be further improved to meet the requirements of accurate extracellular recording. For example, the performance of transducers may be influenced by the bitter substances used as stimulations for cellular measurement. Basically, cell impedance sensor is highly sensitive to changes of ion concentrations and thus fits better with stimuli of low concentrations, while receptor cells require high concentration of taste stimuli. As a result, the response of cell impedance to high concentrations of stimuli may be concealed by the impedance of culture solution.

Although facing many challenges, the biosensing approaches are showing promising prospects and potential applications in many fields, including food quality control, drug discovery, development and formulation and bioengineering of remote communication devices. Biosensors developed based on taste and odor sensation mechanism may provide innovative means to devise novel personal digitalized taste and smell communication devices with high accuracy and speed. Some directions will probably achieve significant progress in the foreseeable future. For instance, the signal transduction mechanisms of gustatory signals and their modification may provide a theoretical foundation for improvement of biosensors performance. New integrated chips may be assembled based on taste cells for applications such as drug development and quality identification of food-derived peptides and traditional Chinese medicine. Understanding of joint chemical space of odorants and tastants and of inter-species differences (i.e., human versus rodents) in chemosensory signal detection may be improved. Enhancers of receptors responses will not only provide more sensitive biosensors but may be used as taste modulating agents. Biomimetic electronic tongue that is able to confirm the predicted bitterness of unknown compounds [19] may be developed based on the array of bitter receptors or cells and makes it possible to construct a fingerprint database for exploring the relationship between the structure and bitterness of the chemical compounds. Fast screening of specific bitter and odorant compounds may provide new platforms for food quality control and monitoring taste of drug candidates during drug discovery and development.

## Abbreviations

ASIC2	acid-sensing ion channel 2
ATP	adenosine triphosphate
ATD	amino-terminal domains
CNG channel	cyclic nucleotide gated channel
EIS	electrochemical impedance spectroscopy
ENaC	epithelial Na channel
FET	field effect transistor
FEDs	field-effect devices
GPCRs	G-protein coupled receptors
GRK2	G-protein-coupled receptor kinase 2
HCNs	hyperpolarization-activated cyclic nucleotide-gated channels
HEK-293	human embryonic kidney 293
hERG	human ether-a-go-go related gene
LAPS	light-addressable potentiometric sensor
MCF-7	Michigan cancer foundation (MCF) -7
MEA	microelectrode array
MFT	2-methyl-3-furanthiol
MOE	main olfactory epithelium
ORs	olfactory receptors
ORNs	olfactory receptor neurons
PCA	principal component analysis
PKD channels	polycystic kidney disease-like channels
QCM	quartz crystal microbalance
RAMPs	receptor activity-modifying proteins
REEPs	receptor expression enhancing proteins
RTPs	receptor transporting proteins
SAMs	self-assembled monolayers
SAW	surface acoustic wave
SPR	surface Plasmon resonance
TCM	traditional Chinese medicine
VFT	Venus flytrap
VNO	vomeronasal organ
